# Supplementary material for: Adverse events reported during weekly isoniazid-rifapentine (3HP) tuberculosis preventive treatment among people living with HIV in Uganda
Source: medRxiv. 2024 Aug 9:2024.08.08.24311632. Preprint. [Version 1] doi: 10.1101/2024.08.08.24311632 (PMC11326332; doi:10.1101/2024.08.08.24311632)
Supplement: Supplement 4 [file media-4.docx]

**Supplement Figure 1.** Most reported laboratory test types and diagnoses made based on test results among study participants taking 3HP.

**
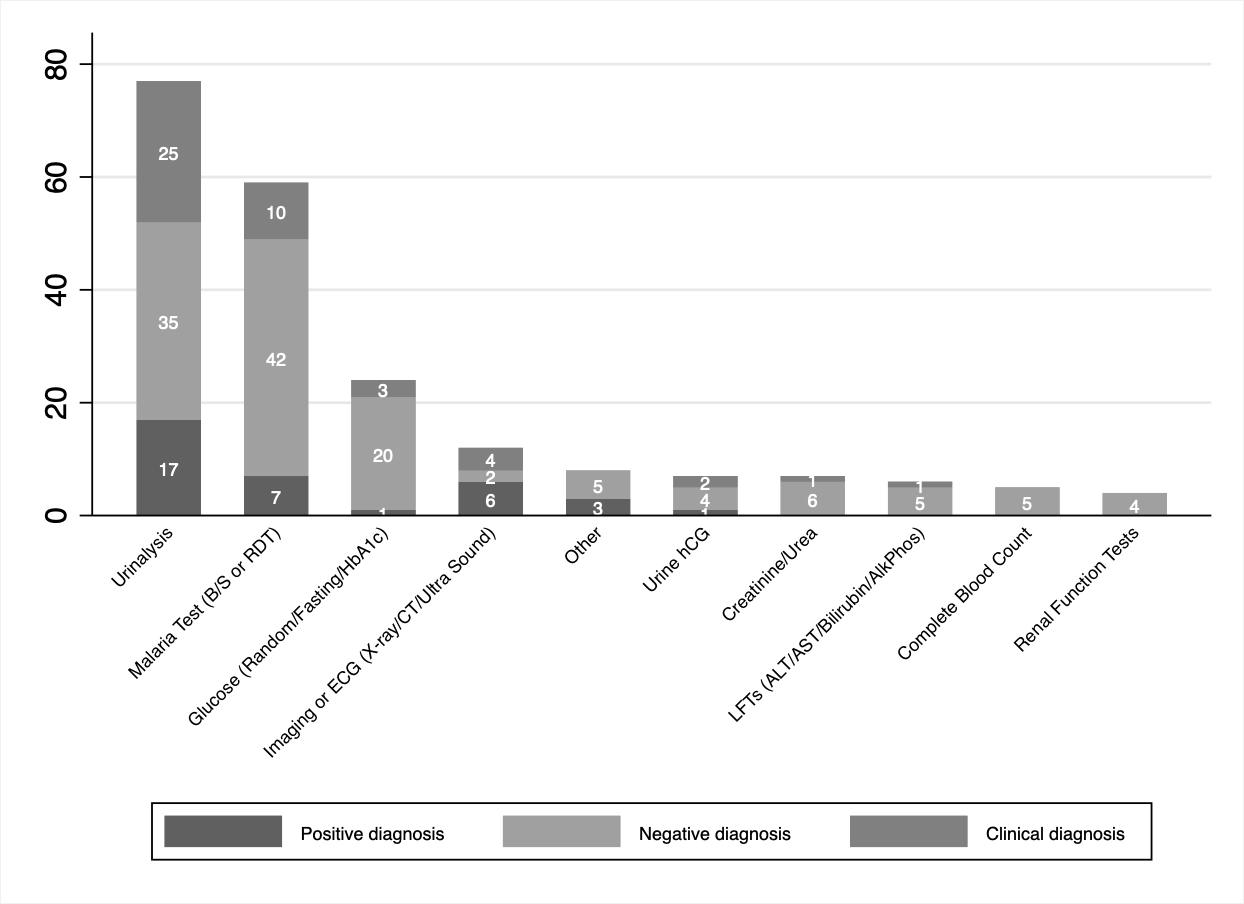
**

ALT=alanine transaminase, AST=aspartate transferase, B/S=blood smear, CT=computed tomography scan, ECG=echo cardiogram, hCG=human chorionic gonadotropin, LFT=liver function test, RDT=rapid diagnostic test
